# Understanding the Transient Nature of STEM Doctoral Students’ Research Self-Efficacy Across Time: Considering the Role of Gender, Race, and First-Generation College Status

**DOI:** 10.3389/fpsyg.2021.617060

**Published:** 2021-01-26

**Authors:** Kaylee Litson, Jennifer M. Blaney, David F. Feldon

**Affiliations:** ^1^Department of Instructional Technology and Learning Sciences, Utah State University, Logan, UT, United States; ^2^Department of Educational Leadership, Northern Arizona University, Flagstaff, AZ, United States

**Keywords:** self-efficacy, research self-efficacy, doctoral student, longitudinal, stability, autoregressive, individual differences, within-person and between-person effects

## Abstract

Developing research self-efficacy is an important part of doctoral student preparation. Despite the documented importance of research self-efficacy, little is known about the progression of doctoral students’ research self-efficacy over time in general and for students from minoritized groups. This study examined both within- and between-person stability of research self-efficacy from semester to semester over 4 years, focusing on doctoral students in biological sciences (*N* = 336). Using random intercept autoregressive analyses, we evaluated differences in stability across gender, racially minoritized student status, and first-generation student status. Results showed similar mean levels of self-efficacy across demographic groups and across time. However, there were notable differences in between-person and within-person stability over time, specifically showing higher between-person and lower within-person stability for racially minoritized and first-generation students. These findings indicate that racially minoritized and first-generation students’ research self-efficacy reports were less consistent from semester to semester. Such results may indicate that non-minoritized and continuing-generation students’ experiences from semester to semester typically reinforce their beliefs about their own abilities related to conducting research, while such is not the case for racially minoritized nor first-generation students. Future research should examine what types of experiences impact self-efficacy development across doctoral study to offer more precise insights about factors that influence these differences in within-person stability.

## Introduction

Self-efficacy (Bandura, 1997) is defined as one’s belief about their ability to be successful in a given domain (Multon et al., 1991; Zimmerman et al., 1992). Of interest in doctoral student training is the development of research self-efficacy, which has been identified as an important part of preparing doctoral students to pursue independent research and academic success. In the context of graduate education, self-efficacy for effectively conducting research has been shown to predict the likelihood of Ph.D. completion (e.g., Litalien and Guay, 2015), scholarly productivity (e.g., Lambie et al., 2014; Hemmings and Kay, 2016), and future academic career success (Kademani et al., 2005). Doctoral students may struggle with developing or sustaining research self-efficacy due to high-stress training environments or the impostor phenomenon, which is when accomplished individuals attribute successes to “fraudulence, fooling others, and luck instead of their own hard work or ability” (Chakraverty, 2020, p. 160). Such a struggle may negatively affect known associated outcomes, including research productivity and academic career success.

### Inequities in Self-Efficacy

The role of doctoral students’ self-efficacy is neither wholly stable nor homogenous across subgroups or level of doctoral study. Studies focusing specifically on *research* self-efficacy are mixed regarding mean differences in research self-efficacy levels between men and women in Ph.D. programs (Brown et al., 1996; Hemmings and Kay, 2016; Feldon et al., 2017) and among minoritized and White graduate students (Santiago and Einarson, 1998). Further, across stages of doctoral study, self-efficacy changes as students are exposed to various influences at differing points in their degree programs (Sverdlik and Hall, 2020). These results parallel academic and science self-efficacy findings in undergraduate STEM programs. Prior cross-sectional research shows that women, students of color, and first-generation college students tend to report lower self-efficacy relative to their male, White, and continuing generation counterparts (Williams and George-Jackson, 2014; Blaney and Stout, 2017).

Not only does self-efficacy during doctoral training differ across subgroups of individuals, associated predictors and outcomes have also shown differences across subgroups of individuals. According to social cognitive theory and social cognitive career theory (Bandura, 1997; Lent et al., 2002), several factors influence self-efficacy beliefs, including successfully completing tasks, vicarious inferences from others’ experiences, feedback/encouragement/support from others, and interpretations of one’s own affective responses. Prior research on doctoral student development has documented inequities across each of these factors. Students from historically marginalized groups within the academy tend to experience less frequent opportunities to publish and disseminate their work (i.e., opportunities to successfully complete tasks) and lower levels of support from faculty and peers (i.e., encouragement/support from others) (Millett and Nettles, 2006; Gardner, 2008; Felder et al., 2014; Griffin et al., 2020). They also are less likely to encounter faculty members from similar backgrounds from which they might identify vicarious evidence of their own abilities (Sheltzer and Smith, 2014; Griffin, 2020) and more likely to struggle with anxiety and depression (Evans et al., 2018; Lilly et al., 2018). Fewer opportunities, role models, lower support, and greater negative affect may negatively impact self-efficacy (e.g., Bandura, 1997), which could in turn negatively impact career aspirations and productivity (e.g., Lent et al., 2002).

To the extent that mean levels of research self-efficacy and factors associated with research self-efficacy show differences across demographic groups, one could hypothesize that the relationships among research self-efficacy and related factors differ across demographic groups. This was reported by Brown et al. (1996), who examined research self-efficacy as a mediator between research training environment and scholarly productivity. They determined that women’s self-efficacy was predicted more substantially by training environment than men’s self-efficacy, but women’s self-efficacy predicted substantially less variance in scholarly productivity than men’s. Similarly, Feldon et al. (2017) identified significant mean differences in research self-efficacy by gender, but found that self-efficacy was a significant predictor of scholarly productivity only for men. Thus, self-efficacy was more influential in predicting scholarly productivity for men than women. Hemmings and Kay (2016) identified differences in the measurement model underlying research self-efficacy as a latent construct between men and women, with self-assessment of skills regarding engaging literature playing a larger role for women than for men. Although studies examining multivariate model differences across racial/ethnic groups were not identified in our review of the literature, gender comparison studies consistently detect these differential sets of relationships between self-efficacy and other variables between men and women.

### Understanding Self-Efficacy Over Time

Especially relevant to our study, when evaluating self-efficacy over time, emergent inequities become more complex. For example, prior scholarship suggests that undergraduate women’s academic self-efficacy is lower than men’s at the outset of college but does not differ from men’s self-efficacy at graduation (MacPhee et al., 2013), the relationship between science self-efficacy and future STEM achievement showed no gender differences (Jungert et al., 2019), and racially minoritized (RM) students’ science self-efficacy was related to the pursuit of a future STEM career 4 years after graduation (Estrada et al., 2018). Collectively, these findings suggest that both the nature and impact of self-efficacy change over time. Thus, findings from existing studies of self-efficacy may largely be contingent on time (e.g., MacPhee et al., 2013), suggesting that relying on data from one or two time points is insufficient. Instead, the moment in which self-efficacy is assessed is measurably important to more directly understand whether, when, and how differences in self-efficacy manifest during graduate education.

When it comes to self-efficacy among Ph.D. students specifically, little work has been devoted to evaluating self-efficacy as it develops over time. Much of the prior work on the longitudinal nature of doctoral self-efficacy has examined self-efficacy within one or two time points, typically at the beginning and end of an educational program (e.g., Zajacova et al., 2005; Paglis et al., 2006). Thus, little is known about the long-term stability and development of self-efficacy during graduate training over time. In particular, understanding how research self-efficacy develops throughout one’s Ph.D. program is important, because doctoral training requires scholarly productivity and publication both as a skill development exercise and as a means of making oneself marketable for postdoctoral employment opportunities (Ehrenberg et al., 2009). To the extent that research self-efficacy influences scholarly productivity, better understanding its mechanisms could permit faculty and practitioners to provide targeted support fostering self-efficacy among students at critical points in their training.

Understanding the longitudinal progression of self-efficacy is further important because it can impact the interpretation of relationships found among self-efficacy and other variables (e.g., the findings about gender differences in multivariate mediation and models discussed previously; Brown et al., 1996). From cross-sectional models, it is impossible to determine the extent to which observed differences in model parameters are attributable to individual differences, subpopulation differences, or an amalgamation of the two. For example, the findings of Brown et al. (1996) and Hemmings and Kay (2016) lack a sufficient number of measurement points to determine whether self-efficacy is influenced by predictors above and beyond its own prior influences. Feldon et al. (2017) engaged a substantial number of measurements across a year of doctoral study for several variables, but they evaluated research self-efficacy measures at only one time point. Ultimately, the interactions between training environment, research self-efficacy, and desirable outcomes of doctoral education (e.g., degree completion, scholarly productivity) are inherently linked to time, yet the development of research self-efficacy as it changes moment-to-moment over the course of time is essentially unknown. Since relationships among self-efficacy and relevant constructs require time-specific precision—especially with regard to group differences—for the field to engage practices likely to enhance student outcomes and equity across groups, the present study aims to rigorously evaluate the progression of research self-efficacy as it develops over time.

### Objectives and Research Questions

This study examines two modes of research self-efficacy stability across the first 4 years of Ph.D. students’ doctoral programs, focusing on doctoral students in the biological sciences. The two types of stability are between-person stability across time (a general, trait-like stability) and within-person stability between consecutive time points (a specific or state-like stability). Specifically, we examine differences in stability across demographic groups, comparing RM students to White and Asian students, first-generation college students to continuing-generation students, and women to men. The following questions frame this study:

1. What proportion of research self-efficacy across time reflects stability *between persons*?
2. To what extent does research self-efficacy from one academic semester predict research self-efficacy for the next semester (i.e., stability *within persons*)?
3. Does the between-person stability of research self-efficacy vary across RM status, first-generation student status, or gender?
4. Does the within-person stability of research self-efficacy vary across RM status, first-generation student status, or gender?

## Materials and Methods

### Participants

The present study draws on data from a larger National Science Foundation-funded project on doctoral students’ research experiences and skill acquisition in the biological sciences. The sample includes *N* = 336 students from 53 institutions who began their doctoral programs in the biological sciences in fall 2014, with an average number of participant per institution of 6.34 (SD = 5.69). Across gender, *n* = 200 students identified as women, *n* = 134 identified as men, and *n* = 2 students identified as other gender identities. Across RM status, *n* = 59 were RM students, *n* = 271 were White or Asian students, and *n* = 6 did not report race nor ethnicity data. Across generation status, *n* = 96 were first-generation students, *n* = 235 were continuing generation students, and *n* = 5 did not report data on parental college education.

### Measures

#### Demographic Variables

Students completed demographic questions about their race, ethnicity, gender, and parents’ education level at the onset of the study. To measure race and ethnicity, students selected one or more of the following: American Indian or Alaska Native; Asian or Asian American; Black or African American; Latino/a; Native Hawaiian or Other Pacific Islander; White. Responses were aggregated to create a measure of RM status where students who selected only a White and/or Asian identity were coded as majority and all other students were coded as RM (*0 = majority, 1 = RM*). To assess first-generation college status, students reported the highest degree obtained by their parent(s). Those who had no parent with a college degree were coded as first-generation (*0 = continuing generation, 1 = first generation*). Students reported their gender identity (*0 = woman, 1 = man, 2 = non-binary or other*). Because few students indicated a non-binary gender identity, we treat gender as a dichotomous variable in analyses that follow.

#### Research Self-Efficacy by Semester

Biweekly surveys asked participants to respond to the question “On a scale of 1 to 7, how confident do you feel in your ability to perform [research]?” where *1 = not at all* to *7 = very highly*. The bi-weekly research self-efficacy reports were averaged for each individual per academic semester, resulting in three occasions of measurement per year: fall semester (S1), spring semester (S2), and summer semester (S3). Reports from winter break were excluded from semester averages because winter break is between two semesters and includes a large chunk of time away from school work. A total of 12 measurement occasions were created from 100 biweekly reports of research self-efficacy.

To determine the validity of the semesterly measure of research self-efficacy, correlations between the spring semester measure of research self-efficacy and an annual measure of research self-efficacy were evaluated. During the spring semester across years, participants received the Research Experience Self-Rating Survey (Kardash, 2000), which asked them to self-rate their abilities to perform each of 10 research related tasks (e.g., To what extent do you feel you can observe and collect data?) on a 5-point Likert scale from *1 = not at all* to *5 = a great deal*. Correlations between the semester and composite Research Experience Self-Rating Survey annual measures within years were moderate, ranging from 0.51 to 0.57 across years. These correlations are similar in magnitude to correlations of the annual measures across time (range 0.36 to 0.66), lower than the correlations between the semesterly measures over time (range: 0.54 to 0.83), and higher than the annual items correlated with semesterly self-efficacy measures (range: 0.30 to 0.50).

### Procedures

Students were recruited in two ways: first, the research team contacted the department chairs and program directors of the largest 100 Ph.D. programs in the biological sciences as well as public flagship and minority serving universities (e.g., Hispanic-serving institutions) that had Ph.D. programs within the subfields of interest. Program directors and department chairs were given recruitment materials and asked to disperse the materials to incoming Ph.D. students. Additionally, participants were recruited by the research team sending emails to listservs, including the American Society for Cell Biology and the Center for the Integration of Researcher, Teaching, and Learning Network. This phase of recruitment approached saturation since all students who responded were entering Ph.D. programs contacted in the first phase of participant recruitment. Participants who responded to the research team were screened for study eligibility. Participants were then given informed consent and told of the expectations for continuing in the study. All students who participated received a $400 annual incentive.

Biological sciences was selected as the single, target discipline for several reasons. First, it reduced the potential for cultural or structural differences between individual STEM disciplines to obscure or bias observed trends. Second, it represents the largest disciplinary area within the broader life sciences, with total awarded doctorates numbering 8,702 out of the 12,781 life sciences Ph.D. degrees granted in the United States in 2019 (National Center for Science and Engineering Statistics, 2020). Third, the biological sciences also represent the most gender-equitable (51.7% woman) and ethnically diverse STEM subfields by PhDs awarded (19.5% RM respondents as a pooled group).

### Analysis

Analyses were conducted in M*plus* version 8.4 (Muthén and Muthén, 1998–2019). We accounted for the data structure of students nested within universities in all analyses by using the M*plus* command, type = *complex*. Missing data were accounted for by using maximum likelihood estimation with robust standard errors.

**FIGURE 1:**
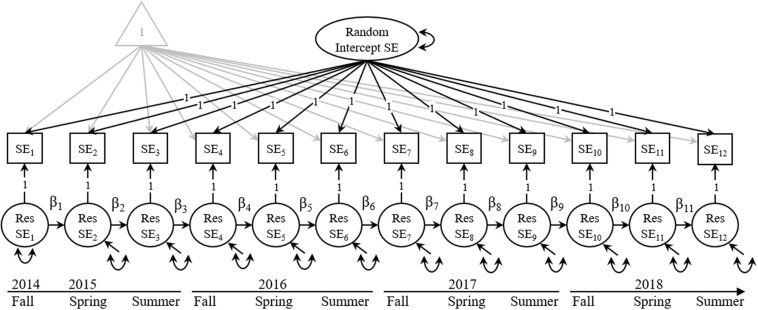
Univariate random intercept autoregressive model of research self-efficacy. SE = research self-efficacy; Res SE = the residual after accounting for the random intercept component of research self-efficacy. The path analysis includes latent variables represented as ovals, manifest variables represented as rectangles, and constants (e.g., means and intercepts) represented as a triangle. Single-headed arrows represent the regression pathways in the model, and double-headed arrows represent variances and/or covariances. Variances of the manifest variables are set to 0.0 to estimate the variance of the Res SE factors. No correlations were modeled.

For the first and second research questions, we utilized a random intercept autoregressive model (see Figure 1), a univariate structure of the random intercept cross-lagged panel model (Hamaker et al., 2015), and similar to single-indicator latent state-trait models (Kenny and Zautra, 1995). The rationale for using this model was to simultaneously disaggregate between-person variance from within-person variance, allowing us to more clearly evaluate stability. The between-person component can be evaluated by examining the variance due to the random intercept factor [similar in concept to evaluating the proportion of trait variance latent state trait models (Steyer et al., 2015)], with higher proportions of variance reflecting greater between-person stability across time. The within-person component was evaluated by examining the autoregressive paths between consecutive time points. Specifically, the autoregressive paths can be interpreted as the “degree by which deviations from an individual’s expected score on *y*… can be predicted from preceding deviations from one’s expected score on *x*… while controlling for the individual’s deviation of the preceding expected score on *y*” (Hamaker et al., 2015, p. 105). In other words, after accounting for each individual’s expected score using the random intercept factor, autoregressive parameters are modeled among the deviations from the expected score. The autoregressive parameters can be interpreted as within-person effects after parsing out between-person variance, and their interpretation differs from a standard autoregressive model. Both the between-person and within-person aspects of stability play an important role in understanding the progression of research self-efficacy as it develops across time.

For the third and fourth research questions, we evaluated multigroup versions of the random intercept autoregressive models. We separated groups by gender, RM status, and first-generation student status. We examined differences in β-coefficients across groups using M*plus* model constraints. We further evaluated differences in the estimated variance of the random intercept factor across groups using model constraints. When models were fit to the entire sample, the full model fit the data well (χ^2^ = 140.94, df = 54, *p* < 0.001, RMSEA = 0.07, CFI = 0.97, TLI = 0.96). Model fit statistics for all analyses are presented in Table 1.

**TABLE 1:** Model fit information.

Model	χ^2^	df	*p*	RMSEA	CFI	TLI
RI-AR	140.94	54	0.00	0.07	0.97	0.96
RI-AR across gender	251.93	108	0.00	0.09	0.96	0.95
RI-AR across first generation status	258.94	108	0.00	0.09	0.96	0.95
RI-AR across racially minoritized status	256.94	109	0.00	0.09	0.96	0.95

*RI-AR, random intercept autoregressive model.*

## Results

### Descriptive Findings

**FIGURE 2:**
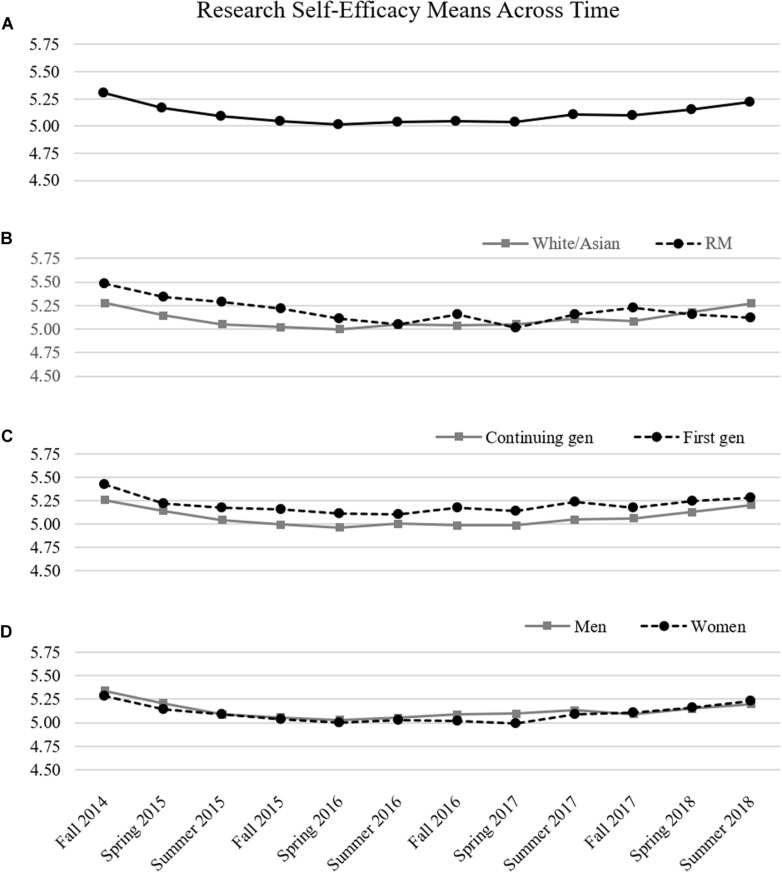
Research self-efficacy means across 4 years of doctoral training. **(A)** Means of research self-efficacy across time for all participants. **(B)** Means are separated across minority status. **(C)** Means are separated across generation status. **(D)** Means are separated across gender.

Means of self-efficacy across both semester and demographic groups are shown in Figure 2. Overall, research self-efficacy means decreased and then increased across time for all students, with the shape of research self-efficacy exhibiting a flat U-shape. We examined pointwise mean differences across groups by using model constraints. Results showed no significant mean differences within time across any demographic groups.

### Research Question One: Between-Person Stability

To first evaluate the stability between-persons, we examined the proportion of variance of each observed variable that was attributed to the random intercept component (i.e., trait influences). The random intercept factor accounted for an average of 47% (range: 0.29 to 0.56) of the observed variable variance across all participants included in the sample. Thus, approximately between one-half of the variance in each observed variable is attributed to between-person stability. Table 2 shows that the proportion of variance from the random intercept factor in the overall sample tends to increase across time.

**TABLE 2:** Proportion of random intercept variance from the univariate random intercept autoregressive models of research self-efficacy.

Semester	Overall	Men	Women	White/Asian	RM	Continuing gen	First gen
Fall 2014	0.35	0.55	0.47	0.62	0.53	0.36	0.53
Spring 2015	0.29	0.66	0.39	0.49	0.57	0.31	0.63
Summer 2015	0.40	0.75	0.57	0.62	0.79	0.41	0.83
Fall 2015	0.45	0.86	0.57	0.65	0.85	0.45	0.89
Spring 2016	0.50	0.86	0.65	0.70	0.85	0.52	0.86
Summer 2016	0.49	0.83	0.63	0.69	0.93	0.53	0.87
Fall 2016	0.49	0.84	0.59	0.67	0.99	0.49	0.92
Spring 2017	0.50	0.86	0.58	0.69	0.85	0.51	0.84
Summer 2017	0.56	0.91	0.61	0.75	0.88	0.57	0.89
Fall 2017	0.51	0.85	0.56	0.71	0.73	0.56	0.72
Spring 2018	0.53	0.84	0.58	0.75	0.84	0.56	0.88
Summer 2018	0.55	0.82	0.62	0.73	0.80	0.56	0.82
*Average across semesters*	0.47	0.80	0.57	0.67	0.80	0.49	0.81

*All factor loadings in the analyses were set to 1.0.*

### Research Question Two: Within-Person Stability

**FIGURE 3:**
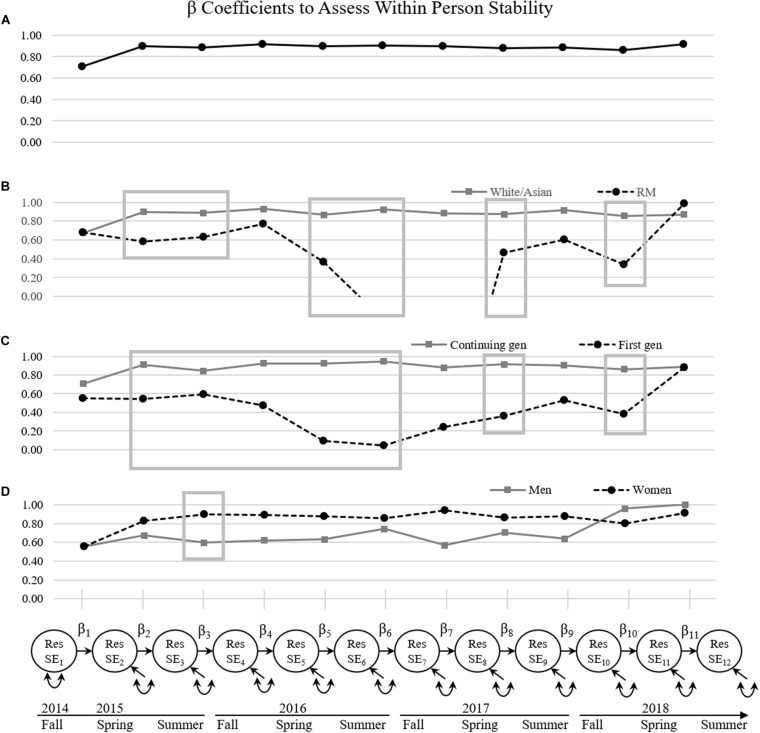
Autoregressive β coefficients to assess stability of research self-efficacy across time and demographics. Res SE = the residual after accounting for the random component of research self-efficacy. Values in the box indicate statistically significant differences across groups in β coefficients. β_6_ and β_7_ in panel **(B)** reflect β values that were less than 0, (–0.26 and –1.84) and are thus not reflected in the graphic. **(A)** β values of research self-efficacy across time for all participants. **(B)** β values of research self-efficacy across minority status. **(C)** β values are separated across generation status. **(D)** β values are separated across gender.

Next, we evaluated the stability of research self-efficacy within persons by examining β coefficients between consecutive time points. Note that within-person stability results should be interpreted within the context of the between-person stability estimates for each group. Greater β coefficient values indicated higher levels of within-person stability while smaller β coefficient values indicated lower levels of within-person stability, and negative β coefficient values indicate that individuals with high deviations from the expected score at time *t* have low deviations from the expected score at the subsequent time point, *t* + 1. Overall, when utilizing the entire sample, the predictive power of preceding deviations from one’s expected score on research self-efficacy was high, with β coefficients ranging from 0.71 to 0.92 (standardized β ranging from 0.63 to 0.90; see Figure 3A). These values reflect a 1-unit deviation from the expected score on research self-efficacy at time *t*, predicting a 0.71 to 0.92 deviation from an individual’s expected score on research self-efficacy at *t* + 1. This suggests high levels of within-person stability. All β coefficient values were statistically significant, showing that the deviation of self-efficacy at preceding time points was a strong predictor of the deviation of self-efficacy at later time points.

### Research Question Three: Group Differences of Between-Person Stability

We evaluated differences in variance attributed to the random intercept component across race/ethnicity, generational status, and gender (see Table 2). White and Asian students had a significantly lower amount of random intercept variance than RM students (0.31 for White/Asian students, 1.12 for RM students, *t*_diff_ = 4.02, *p*_diff_ < 0.001). This finding was further reflected in the lower average proportion of variance attributable to the random intercept factor for White/Asian students (0.67) compared to RM students (0.80). A similar finding emerged when comparing random intercept variance across generational status. First-generation students had significantly more variance attributable to the random intercept factor than continuing generation students (0.91 for first-generation students, 0.29 for continuing-generation students, *t*_diff_ = 2.43, *p*_diff_ = 0.02). The average proportion of variance attributable to the random intercept factor for first-generation students was 0.81 while this value for continuing-generation students was 0.49. Random intercept variance for men (0.83) compared to women (0.35) was not statistically significantly different (*t*_diff_ = 1.46, *p*_diff_ = 0.14). However, the proportion of variance due to the random intercept seems to reflect some differences, with men showing a higher average proportion of variance attributable to the random intercept factor (0.80) compared to women (0.57).

### Research Question Four: Group Differences of Within-Person Stability

Across race/ethnicity, autoregressive estimates differed across years (see Figure 3B). Over time, RM students had significantly lower within-person stability than White/Asian students between spring and summer of year 1 (β_diff_ = 0.31, *t* = 3.09, *p* = 0.002), summer of year 1 and fall of year 2 (β_diff_ = 0.26, *t* = 1.96, *p* = 0.05), spring and summer of year 2 (β_diff_ = 0.50, *t* = 3.09, *p* = 0.002), summer of year 2 and fall of year 3 (β_diff_ = 1.18, *t* = 4.97, *p* < 0.001), spring and summer of year 3 (β_diff_ = 0.41, *t* = 3.67, *p* < 0.001), and fall and spring of year 4 (β_diff_ = 0.52, *t* = 4.11, *p* < 0.001). Notably, between the spring and summer semesters in years 1, 2, and 3, RM students had significantly lower autoregressive values than White/Asian students. For RM students, deviations from their expected value do not predict future deviations to the same extent as White/Asian students. In two cases, deviations from expected values at time *t* negatively predict deviations from expected values at time *t* + 1.^1^ Thus, individuals are deviating from their expected research self-efficacy value in a way that is not (or negatively) predicted by prior deviations, suggesting a lack of within-person stability for RM students.

Similar to results across race/ethnicity, many differences in within-person stability were found across student generational status (see Figure 3C). Continuing generation students had statistically significantly higher within-person stability than first-generation students between spring and summer of year 1 (β_diff_ = 0.37, *t* = 4.51, *p* < 0.001), summer of year 1 and fall of year 2 (β_diff_ = 0.25, *t* = 3.51, *p* < 0.001), fall and spring of year 2 (β_diff_ = 0.45, *t* = 2.52, *p* = 0.01), spring and summer of year 2 (β*_diff_* = 0.83, *t* = 3.54, *p* < 0.001), summer of year 2 and fall of year 3 (β_diff_ = 0.90, *t* = 2.62, *p* = 0.01), spring of year 3 and summer of year 3 (β*_diff_* = 0.55, *t* = 3.36, *p* = 0.001), and fall of year 4 and spring of year 4 (β_diff_ = 0.48, *t* = 2.91, *p* = 0.004). For first generation students, deviations from their expected value did not predict future deviations; thus, individuals were deviating from their expected trajectory of self-efficacy in a way that was not influenced by prior deviations, suggesting a lack of within-person stability.

Unlike race/ethnicity and generational student status, within-person stability was more similar across gender. Only one difference in the within-person stability of research self-efficacy was found. Women had higher within-person stability than men between summer of year 1 and fall of year 2 (β_diff_ = 0.29, *t* = 2.66, *p* = 0.01). No other gender differences were found (see Figure 3D).

Notably, our findings suggest that research self-efficacy was less stable within-person for RM students and first-generation college students relative to their majority and continuing-generation counterparts. However, their between-person stability was greater, indicating more robust trait level effects within minoritized groups.

## Discussion

The present study has significant implications for understanding the ways in which the cumulative experiences of doctoral students from minoritized backgrounds have different impacts than their peers from non-minoritized groups. Specifically, non-RM and continuing-generation students demonstrate a relatively high level of within-person stability in their self-efficacy over time, suggesting that their experiences from semester to semester typically reinforce their beliefs about their own abilities related to conducting research, especially during the second and third years of graduate education. In contrast, RM and first-generation college students show a noticeable drop in the within-person stability of those beliefs on the basis of the beliefs they held in prior semesters, despite mean levels of self-efficacy being essentially equivalent compared to White/Asian and continuing-generation students. This suggests that even if these groups do not differ significantly from other demographic groups in terms of mean values within a given period of measurement, they may be substantially more influenced by recently unfolding events, whether positive or negative. In other words, exploring mean differences between groups in isolation fails to capture the entire story of how self-efficacy evolves. Taken together, these results demonstrate the importance of looking beyond mean differences at select time points to explore how self-efficacy differs across time between and among groups.

This insight expands previous quantitative research that has found very few differences between the experiences of first-generation students pursuing a Ph.D. and their continuing generation counterparts when examining mean differences (Roksa et al., 2018). Indeed, Roksa et al. (2018) noted that while the likelihood of publishing in a peer-reviewed journal did not differ significantly between groups, the year-to-year correlation in number of publications for first generation college students was substantially weaker than for continuing generation students. Similarly, Feldon et al. (2017) found that men and women’s likelihood of publishing a journal article in the first year of their doctoral programs did not differ significantly. However, women’s odds of publishing decreased relative to those of men as the number of hours worked in the laboratory increased. These findings are consistent with related qualitative research on these topics, which has noted important differences in the experiences of students from various demographic groups relative to men, non-RM students, and continuing generation students (e.g., Holley and Gardner, 2012; Felder et al., 2014). Further, examining experiences and perceptions over time provides important convergence across methods and reflects the valuable insights available through longitudinal analyses that emphasizes statistical variability—rather than mean differences—as a key metric.

In addition to further contextualizing prior research, our findings provide new insights into the ways in which identity factors may shape self-efficacy within social cognitive theory (Bandura, 1997). While social cognitive theory has long held that self-efficacy and its predictors can vary across groups as a function of differential experiences, previous research has emphasized mean differences without examining the evolution of both between- and within-person effects over time. Differential susceptibility of research self-efficacy to reflect changes in the training environment highlights the need to avoid a *priori* assumptions that individual responses to training interventions will have universal effects both among persons and at discrete time points. The findings in this study illustrate clearly that experiences during doctoral study differentially impact individuals in relation to the stability of their self-beliefs, regardless of whether or not measured values differ at a given point in time. Given the variation both within and between groups, self-efficacy may be even more malleable than previously understood.

### Implications

Our findings have important implications for both research and practice in doctoral education. In particular, our findings highlight the importance of collecting self-efficacy data across time in a longitudinal manner and evaluating the different facets of the stability of self-efficacy in addition to evaluating simple mean values of self-efficacy. This is particularly important when comparing self-efficacy across demographic groups or other factors. Mean differences may not be present, but that does not mean differences in research self-efficacy do not exist, and such differences may highlight issues of different types of development over time across groups, lack of measurement invariance, or true group differences. While self-efficacy is highly domain-specific, similar principles may apply to self-efficacy across other domains and populations, a topic that warrants future inquiry.

Engaging a mixed methods approach to understanding these phenomena may represent an important next step in research on these issues. Future research should specifically examine the types of experiences and subsequent meanings that participants construct that occur during doctoral study for RM and first-generation students that differentiate them from their non-minoritized and continuing-generation peers. Because individuals are not always aware of which specific experiences affect their self-efficacy over time (Sherman et al., 2009; Walton and Cohen, 2011), such studies could offer more precise insights regarding factors that potentially contribute to or mitigate within-person changes after accounting for between-person group level effects. Such efforts can also take place across multiple disciplines, thereby addressing the limitations of this study’s exclusively quantitative perspective and its locus within a single discipline.

In terms of implications for practice, we identified patterns in how self-efficacy tended to be particularly unstable for first-generation and RM students, providing insight into critical points to develop self-efficacy. Specifically, providing targeted programming to support students during the summers may be important for all students, but especially for RM students and those who are first-generation to college. Structured reading groups, writing support programs, and other professional development programming may all be ways to foster community and provide additional support to students over summer months, which may go a long way in fostering self-efficacy by providing opportunities for students to demonstrate their skills and gain validation.

### Limitations and Future Research

Due, in part, to the lack of diversity within STEM doctoral programs, the present study lacked the statistical power to evaluate the intersectional nature of the dataset. Women, RM students, and first-generation college students are not mutually exclusive groups, and existing literature on self-efficacy and equity in education documents the importance of considering such intersections (e.g., Blaney and Stout, 2017). Future research should examine the intersecting nature of identity as it influences the stability of research self-efficacy. Future research may also evaluate research self-efficacy by semester using a composite scale instead of a single item measure. Further, the present study does not determine cause and effect. By design, this study is a descriptive longitudinal study, and the results should be interpreted within this context. Causal inference would require additional data, theory, and research design that could be examined in future research endeavors.

## Conclusion

STEM graduate education can serve as a means to enhance students’ upward economic mobility (Posselt and Grodsky, 2017) and address societal challenges (Cherwitz and Sullivan, 2002). However, STEM graduate training can only serve this purpose if we attend to inequities across myriad outcomes, including experiential differences that may affect the stability of individuals’ self-efficacy. Social cognitive theory highlights the dynamic role of the structures and functions of graduate education can play in self-efficacy development, influencing belief stability, which may have subsequent derivative effects. As stated by Bandura (2012, p. 32), “forms of self-efficacy are for navigating the journey, not just reaching the destination.”

## Data Availability Statement

The raw data and analysis files used to support the conclusions of this article are publicly available at: https://osf.io/7bk8h/.

## Ethics Statement

The studies involving human participants were reviewed and approved by the Utah State University Institutional Review Board. The patients/participants provided their written informed consent to participate in this study.

## Author Contributions

KL, DF, and JB conceptualized and wrote sections of the manuscript. DF acquired funding and designed the study. KL performed statistical analysis and provided data visualizations. KL and JB wrote the first draft of the manuscript. All authors contributed to manuscript revision, and read and approved the submitted manuscript.

## Conflict of Interest

The authors declare that the research was conducted in the absence of any commercial or financial relationships that could be construed as a potential conflict of interest.

## Footnotes

1 One RM deviation between fall 2016 and spring 2017 was very low (−1.84) and had a much larger standard error (2.24) than other estimates, which led to this difference between RM and White/Asian students being non-significant. This abnormality may be a consequence of the generally lower sample size of RM students, model overfitting, or an event occurrence that impacted RM students’ research self-efficacy stability during this time. It is unclear, given the present data, why this estimate and standard error were abnormal.
